# A user-guided personalization methodology to facilitate new smart home occupancy

**DOI:** 10.1007/s10209-022-00883-x

**Published:** 2022-06-15

**Authors:** S. M. Murad Ali, Juan Carlos Augusto, David Windridge, Emma Ward

**Affiliations:** 1 Department of Computer Science, Middlesex University, The Burroughs, Hendon, London, UK; 2 Department of Psychology, Middlesex University, The Burroughs, Hendon, London, UK

**Keywords:** Smart home, System adaptation, Transfer learning, System personalisation

## Abstract

Smart homes are becoming increasingly popular in providing people with the services they desire. Activity recognition is a fundamental task to provide personalised home facilities. Many promising approaches are being used for activity recognition; one of them is data-driven. It has some fascinating features and advantages. However, there are drawbacks such as the lack of ability to providing home automation from the day one due to the limited data available. In this paper, we propose an approach, called READY (useR-guided nEw smart home ADaptation sYstem) for developing a personalised automation system that provides the user with smart home services the moment they move into their new house. The system development process was strongly user-centred, involving users in every step of the system’s design. Later, the user-guided transfer learning approach was introduced that uses an old smart home data set to enhance the existing smart home service with user contributions. Finally, the proposed approach and designed system were tested and validated in the smart lab that showed promising results.

## Introduction

Smart homes gain popularity every day due to the smooth, desirable automation services they provide. User activity recognition is a vital part of the smart home system to precisely provide the necessary services. Currently, scholarly work shows that a sensor-based smart home commonly uses data-driven or knowledge-driven technology [32]. Data-driven intelligent home technology works well if a sufficient amount of data is available. By increasing the dataset, performance increases proportionately, but only to a certain point [42].

Imagine a scenario where an elderly user decides to continue living independently. Despite concerns about safety and proper care, the user’s family decides to accommodate the user in a new smart home where their daily living activities, such as personal hygiene and food preparation, are facilitated by technology. The same home can also provide advanced functionality such as fall detection and other safety and security services. One question that might arise in this scenario, however, is whether the chosen technology will provide the needed help to the user immediately after they move into the new house. A smart home might not be able to answer this question due to its reliance on a large amount of data. This dependency upon data creates the ***cold-start*** problem. A smart home needs sufficient amount of data to recognize, understand and predict user behaviour and to provide the required services [22]. This data dependency of the smart home may increase the user’s family concern about their independent living, especially when they first move in, causing them to underestimate the long-term capabilities of the smart home.

**Fig. 1:**
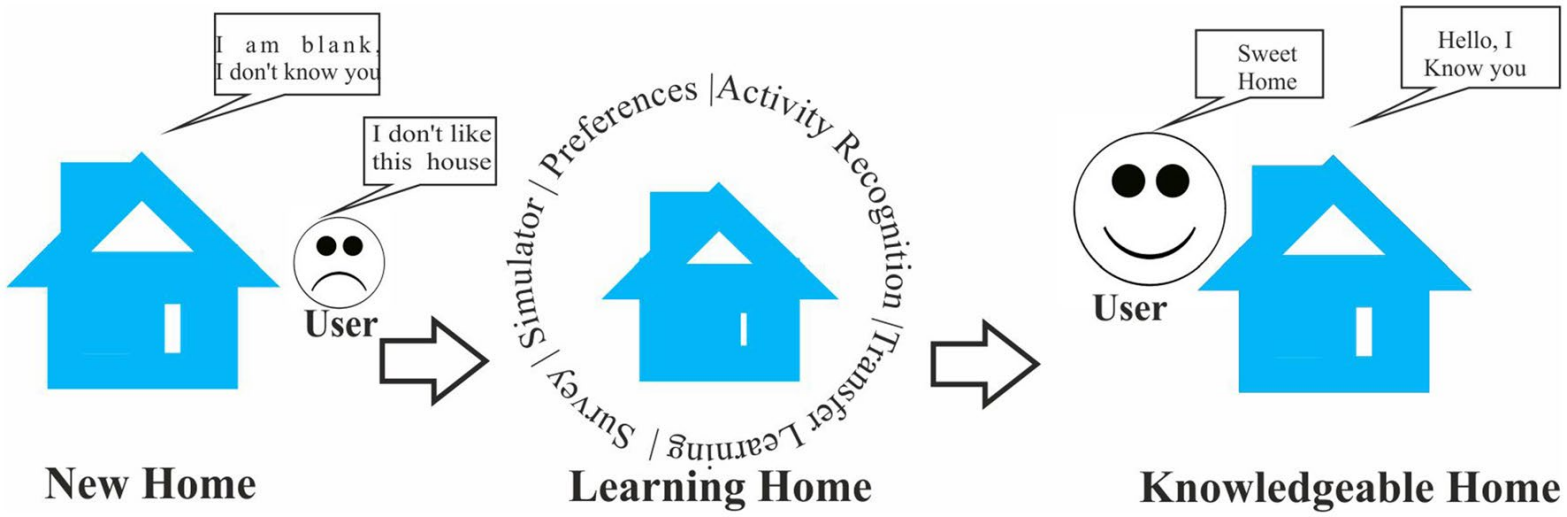
Conceptual illustration of the project

The paper proposes and validates an integrated system that can provide user smart home services as soon as the user starts living in the new house to mitigate the ***cold-start*** problem. Figure 1 shows the conceptual illustration of the project.

Firstly, the useR-guided nEw smart home ADaptation sYstem (READY) is developed using a user-centric approach that brings together three methods: survey, simulation, and activity recognition- to tackle the cold-start problem. Secondly, the User-guided Transfer Learning (UTL) approach is suggested as a complementary method to acquire knowledge from the old smart home and to enhance the understanding of the new home.

Section 2 aims to investigate related work that has been done for smart home adaptation. Literature review emphasizes user-centric, simulation, activity recognition, and transfer learning approaches for smart home adaptation. After identifying any gaps in these areas, we will then examine any previous work in this area. This section certifies the novelty of the project.

The Methodology to be used for the project is described in Sect. 3. The user-centric approach considered for the project aims to keep the user at the centre of the development process, thereby ensuring a higher chance of system acceptance.

READY is a method to integrate three systems together and build a new system that provides a user with smart home services as soon as the user starts living in the home. The development process begins with gathering insights into user behaviour as they perform their daily activities by interview. Later, each of the components will be added based on the requirements and a tailored system will be designed as per the user’s requirements. The evaluation of READY is an iterative process. Each component iteration added to the system is validated by the user before proceeding to the next one. Section 4 explains more details of the READY method.

The complete system testing appears in Sect. 5, where details of the testing house and participants are explained. As mentioned previously, each unit of the system is individually developed and validated. However, in Sect. 5, the system is tested as a whole using Context-aware systems testing validation (COATI) [14] approach to gather insights from the different users. The purpose of this step is to identify potential issues and benefits that may not be evident when the entire system is tested by different users. Details of the testing process, results and also feedback from participants will be provided in this section. The results of the testing are discussed in Sect. 5.3.

Finally, Sects. 5.4 and 6 describe the evaluation and conclusions of the research work reported in this article. They mainly comprise the results of the experiments received from validation and pinpoint the benefits of the project and the challenges faced, also suggesting future directions to ensure continued research in this area.

## State-of-the-art

In this section, we explore the current state of new smart home adaptation. This literature review will focus primarily on activity recognition, which serves as a foundation for all smart home research. First, we present several closely aligned approaches that have been offered as solutions to the new intelligent home adaptation problem. Then, we introduce and justify the proposed approach against those that currently exist. This literature review is an expanded and improved version of a preliminary version published in an earlier article [7].

Activity recognition is the basis upon which a smart home is able to provide personalized automation services. Typically, activity recognition is approached in one of two ways-with knowledge-driven [24, 33] or data-driven approaches. Knowledge-driven [37] approaches leverage prior domain knowledge to model user activities by way of knowledge acquisition, formal modelling, and knowledge presentation. Activity recognition and prediction, in this approach, are driven by logical reasoning tasks such as deduction, induction, and abduction. Knowledge-driven methods are advantageous in that they are easy to understand and logically elegant. Perhaps more importantly, because they draw on prior domain knowledge, they enable users to obtain services right away, thus circumventing the infamous cold-start problem [15]. In contrast to knowledge-driven models, data-driven models learn from datasets that contain user behaviours via data mining and machine learning techniques. These models use probabilistic or statistical methods to overcome data uncertainty issues.

Based on the categorization proposed by Jebara [30], data-driven approaches can be further divided into two distinct classes: generative and discriminative.

Generative algorithms use probabilistic models to build a complete representation of the input data [31]. With an adequate model of the input data, generative algorithms can model the probability distribution for a given dataset, from which they can then predict new data points. The naive Bayes classifier [39] exemplifies this approach to activity recognition, as it uses the joint probability distributions of variables to predict new data. Another popular generative approach is the Hidden Markov Model (HMM), which models probabilities based on transitions from a previous state to a current state [34]. Although HMMs can manage temporal information efficiently, they demand large amounts of data in order to construct a complete probabilistic representation.

Unlike generative approaches, discriminative approaches use conditional probabilities among variables to distinguish between classes of data. These approaches are unconcerned with the distribution of the input data and instead focus on classifying activities based on decision boundaries. For instance, the nearest-neighbor algorithm compares training datasets and determines the most closely matched sequences [17]. Similarly, decision trees are used to learn logical descriptions of activities from complex sensor readings [35, 41].

With these approaches, the main challenge is to find hard data points (i.e., those closest to the boundary). These data points, known as support vectors, are used in the well-known Support Vector Machines (SVM) machine learning technique [18]. SVMs are established and well-known classification methods that classify data in a non-probabilistic way. Other popular algorithm types include Artificial Neural Networks (ANN), which offer various advantages for both activity recognition and learning processes in smart home applications [31]. Popular ANN applications in deep learning include recurrent neural networks, deep feed-forward networks and convolutional neural networks. These algorithms perform better than SVM, NB, and HMM [11, 27].

There are other approaches that do not clearly fall into the discriminative or generative category. For instance, the Independent LifeStyle Assistant (ILSA) uses rule-based and statistical models to make classifications. Similarly, the Learning Frequent Pattern of User Behavior System (LFPUBS) uses rules of association to find the most frequent patterns to determine and implement event condition action rules to detect patterns in real time [26].

We ruled out alternative approaches such as end-user development [19] where users are asked to use a pseudo-programming interface to create automation rules given most common users tend not to be interested, or capable of, getting involved with the system at that level. Our proposed approach only requires lifestyle and preferences feedback from the users at the beginning as a starting point to address the cold start problem and then the system learns from the user daily activities. There are other complementary strategies recently proposed to give participation to the user in influencing system behaviour such as [10, 38].

In all of the aforementioned approaches, data is the primary fuel powering the technology. This presents somewhat of a dilemma, as new smart home users may expect to receive services as soon as they move into a home, while the smart home needs data in order to provide these users with services. To address this issue, researchers have created innovative approaches, such as transfer learning, where a system can draw on user data from previous tasks to improve the performance of new tasks, thus solving the problem of missing training data [23].

Transfer learning represents another approach to solving the data scarcity problem where an old smart home is used as a source and a new smart home used as a target. The no data-target domain category is similar to the problem proposed here. Unfortunately, research has been limited in this area. At present, only one approach proposed by Chiang and Hsu [20] illustrates a possible new smart home adaptation process. The method accommodates a user in the new smart home, where an intelligent system is built for a smart home in a laboratory environment. As data is collected, a transfer learning approach is used to pass the data to the smart home [20]. Chiang et al. [21] proved that without any target data (i.e., no data), the amount of transferred knowledge is insufficient, but it can be increased by using a small amount of labelled data.

Azkune et al. [15] have proposed a new approach in which a survey is distributed among target users in order to determine how they perform daily activities within a smart home. The survey data is then processed by synthetic data generator tools for an arbitrary number of days to generate a labelled activity dataset. However, this approach does not provide any synthetic data evaluation. Thus, the created dataset is used only for modelling and recognizing user activity in a smart home. If the dataset does not apply to the user, the process does not suggest any alternatives.

This paper’s main purpose is to address the new smart home adaptation problem, which has not yet been thoroughly explored in the literature. We first consider a novel user-centric method in which several iterations take place, each beginning with user feedback. Then, we describe the method of designing, developing, and validating this method. In the final phase, we run a smart home simulation in which the user takes an active role in testing and familiarizing themselves with the home.

Importantly, this research offers key advancements to the literature on smart home technology. First, the user is involved at each stage of the development process, which may increase the odds of user uptake and satisfaction. This may be especially important for users who have anxiety over unfamiliar devices (e.g., sensors, interfaces) or those with physical or mental limitations (e.g., dementia) that make learning new technologies a lengthier process [8]. Second, unlike other approaches, the simulator in this method leverages both the transfer learning approach and user feedback to generate data for evaluating the model that closely resembles data generated by a real smart home. Taken together, these contributions make the READY system a notable and influential addition to the literature on smart home technology. Moreover, the approach can be applied to essential contexts, i.e. healthcare, which impacts well-being and health.

## Methodology

**Fig. 2:**
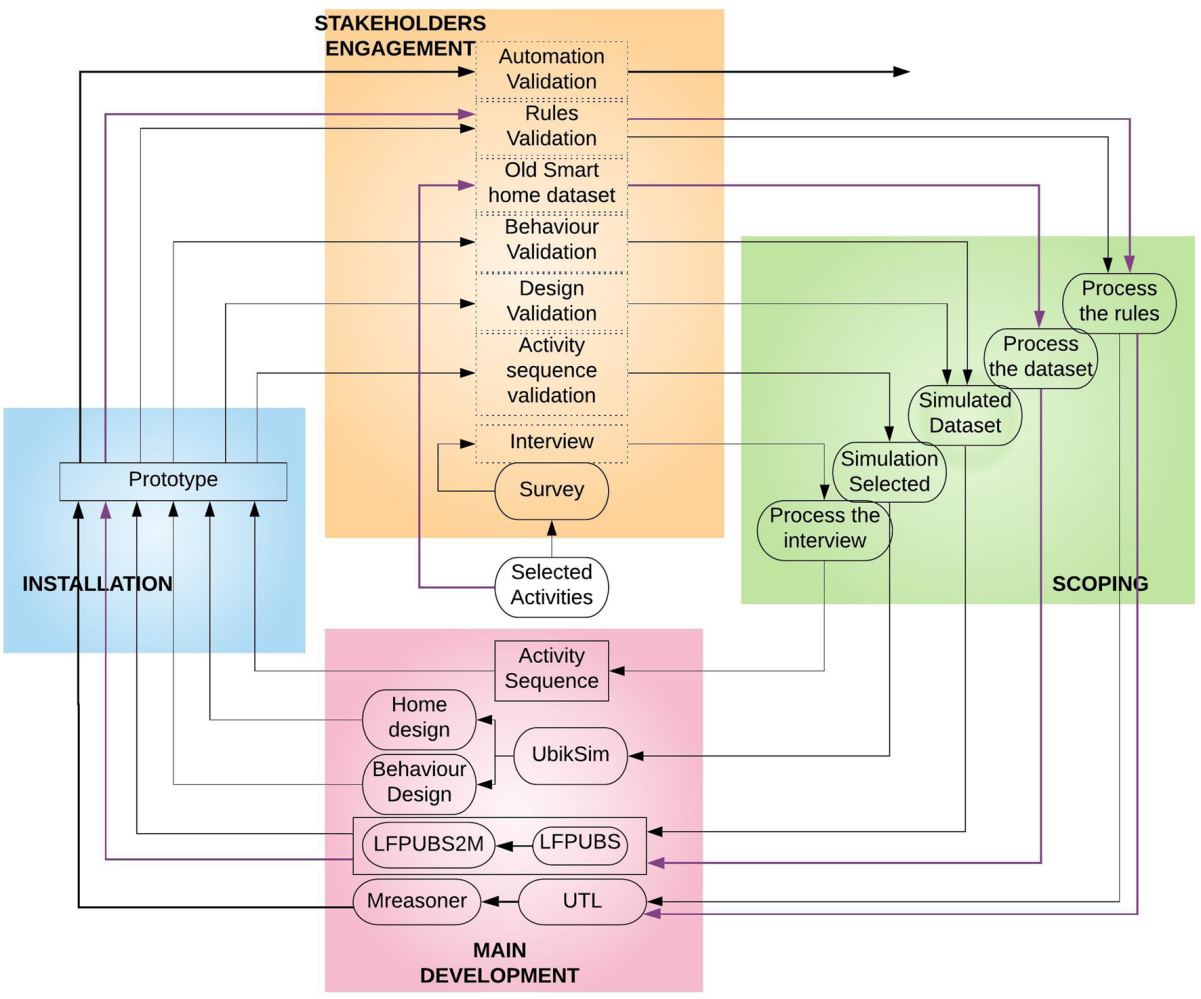
Methodology process

This section describes the methodology used for this project. The User-Centric Intelligent Environment Development Process (U-CIEDP) is relevant for this project as it situates the user at the centre of the development process. U-CIEDP has some unique features which convince us to consider the framework. For example, frequent stakeholder involvement throughout the project and the life cycle of the sensing system is emphasised during design and installation. Several significant developments successfully applied the U-CIEDP framework. For example, recently, Quinde et al. [36] used U-CIEDP to develop context-aware solutions to support the personalisation of asthma management plans. Augusto et al. [12] applied U-CIEDP for the POSEIDON (PersOnalised Smart Environment to increase Inclusion of people with DOwn’s syNdrome) project, which aimed at helping people with Down’s syndrome in smart environment. In POSEIDON, U-CIEDP used as an iterative co-design methodology that involved all the stakeholders.

Figure 2 shows how the research project fits with the U-CIEDP framework and represents the incremental development of a new house adaptation system where every iteration was accomplished by user observation. The method consists of four stages: Stakeholders Engagement, Scoping, Main Development and Installation. The stage *Stakeholder’s engagements* shows the interactions that took place with the stakeholders. *Scoping* captured and conceptualized all of the ideas gathered through stakeholder interaction. The *Main development* is the section where multiple systems were integrated, and READY was developed based on the requirements explained in the next section. Finally, the components developed for main development are installed to ensure that they meet the needs of stakeholders in the *Installation* section.

It is important to explain each of the iterations in-depth to enable the reader to understand the project comprehensively. The project began with the *selected activities*. Activities were selected based on the smart home services the user expects. *Survey* questions were prepared for an interview based on the selected activities. In the interview, the user was asked to describe how they perform the daily living activities. The details of the activities and questionnaires will be explained further in Sect. 4.1. The users’ answers were processed, and *activity sequences* were created as the first step in the development of a READY prototype. Afterwards, sequences were validated with each user to ensure that they meet the users’ needs.

The technical team then determined the appropriate tools required to translate the activity sequences into data. To convert sequences into data, several simulation tools are available, but none meet the project requirements since most of the simulation [28] focuses on generating datasets to test machine learning algorithms. On the other side, the proposed approach uses real data for the simulation. After some conversion, the data is implemented in the system, which provides services to the user. Therefore, it is vital to carefully choose the simulation so that the significance of each user’s needs is not lost. In the initial analysis, UbikSim [40] was selected, although it had not been developed specifically for smart homes. Despite its shortcomings, the software’s core design was close to our requirements, making it easy to use. In Sect. 4.2, we describe how READY was customized to make UbikSim work for the project. We can design a virtual house and an avatar (which represents a virtual user) once the prototype is ready. Furthermore, the prototype is capable of generating simulated data.

Assuming the simulation dataset generation has solved the “cold-start” problem, we needed a system to verify if the simulation dataset reflects accurate information about user behavior. The LFPUBS learning system by Aztiria et al. [16] was used for the READY approach. It elicited knowledge from the underlying dataset and represented the discovered knowledge as a list of patterns. The representation of patterns helps the developer to form an in-depth understanding of the surfaced knowledge, which is vital to the success of the overall system. Section 4.3 explains the features of the LFPUBS system in detail.

Usually, the learning system catch up with the reasoning system. A framework of the reasoning system is available [13]. The project achieves its eventual goal of ensuring that the user receives automation services. Ibarra et al. [29] introduced MReasoner that is influenced by Galton and Augusto [25]. M reasoning system is capable of handling causality within context-aware systems, such as a modern smart home. A benefit of using the MReasoner is that both MReasoner and LFPUBS were developed based on the ECA (event-condition-action) paradigm; that is why they are pretty close to each other in syntax. Furthermore, with the support of an LFPUBS2M translator [9], LFPUBS data can not be passed directly into MReasoner. We, therefore, added LFPUBS2M translator to the prototype.

The User-guided Transfer Learning (UTL) approach helps to increase the acceptance of the current rules generated from the *simulation dataset* and allows the developer to create an updated version of rules with the user guide. The old smart home dataset is the main component of this approach, where, similarly, the old smart home dataset (old dataset) is used to generate the rules, which can be called old rules. The old rules are used to modify the current rules (simulated rules), and the user guide is also provided to the developer for this modification process. A description of how the rules were generated and modified is given in Sect. 4.4.

Following the U-CIEDP approach there were iterations of increasing stakeholder involvement. All of the components mentioned in Fig. 2 have been described above. Five users were involved in system testing in Middlesex University Smart Lab. Next, twelve participants from a wide range of fields joined a live online event to test smart home technology. During the project, all University procedures were followed for ethical clearance, and all testing and validation activities were formally assessed and approved by the ethics committee. The following section explains the useR-guided nEw smart home ADaptation sYstem (READY), the paper’s main contribution.

## useR-guided nEw smart home ADaptation sYstem (READY)

**Fig. 3:**
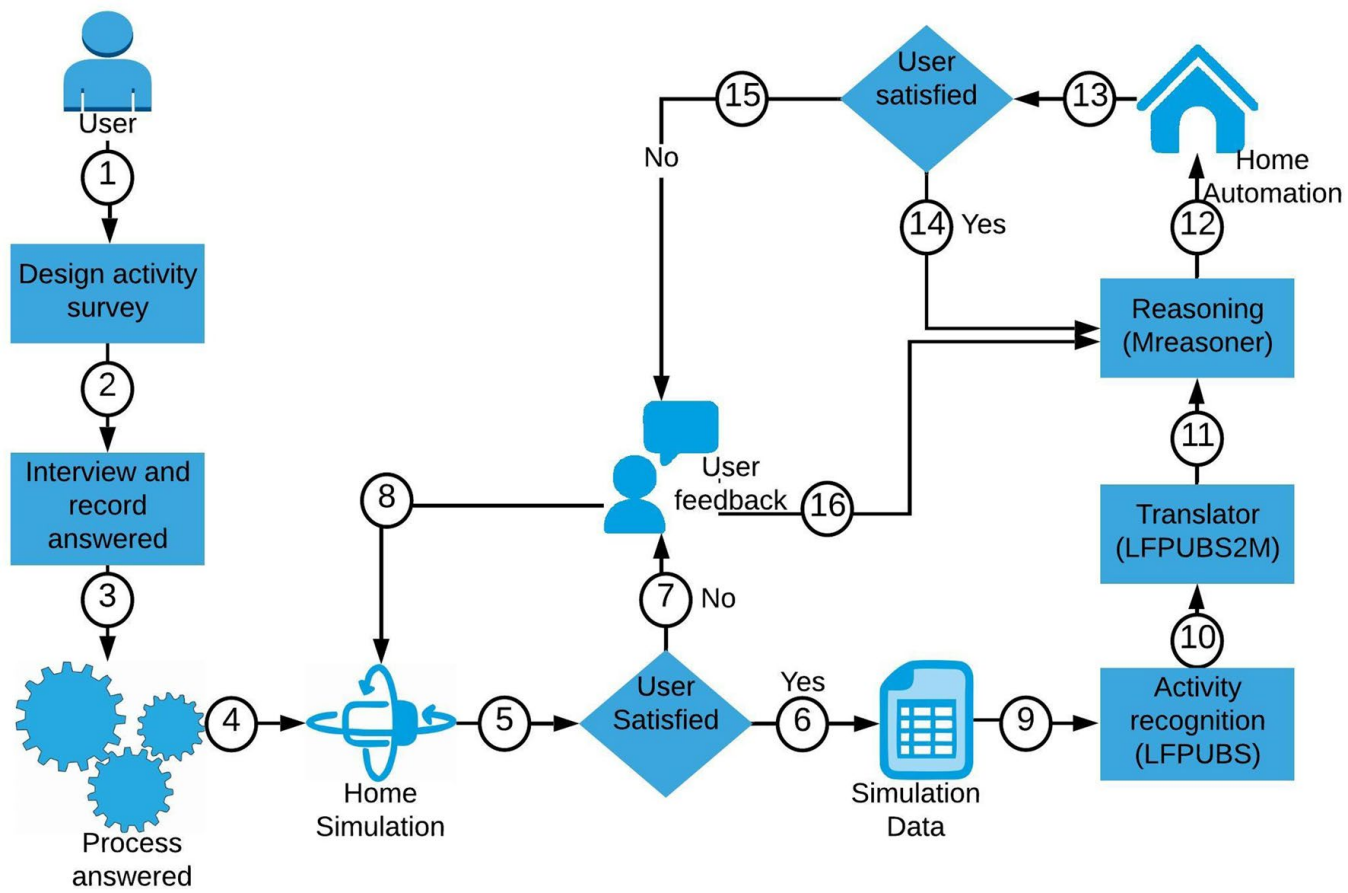
READY system architecture

This section describes the READY method. Figure 3 shows the conceptual architecture of the system (numbering will be used further down to explain the process). READY aims to provide a user with smart home services as soon as they start living in a house. READY is an integrated system that brings together four separate approaches: survey, simulation, activity recognition and transfer learning. As mentioned previously, adopting a U-CIEDP approach allowed the system to evolve as a natural consequence through several iterations before building the final system.

READY is the critical element of this project. The first version of READY is important because the data collected from the users is very raw. Data is processed by developers and entered into the system, but errors made at this point will affect the final version of READY. Therefore, the initial interview should be conducted face-to-face with the user in order to ensure that the interviewer is able to retrieve the necessary knowledge without misunderstanding. The U-CIEDP approach ensures that the next iteration of READY development will only occur if the user has agreed with the current one. User responses are processed and converted in sequence to facilitate the next step. The data processing technique utilised in this study is explained in Sect. 4.1.

A simulation is a tool well-suited to transform an interviewed answer to a daily activity dataset. Hence, simulation was added to the READY method. A simulation mimics a real smart home where data is generated by an avatar when it moves within the smart home and either actively or passively interacts with the virtual sensors. Section 4.2 contains details of the simulation.

Activity recognition is a well-known approach for the extraction of human behaviour from a dataset. The final aim of this project is to provide the home automation service to the user. So, it is essential to understand user behaviour before automation. It is for this reason that READY includes activity recognition and automation tools. Section 4.3 contains further details of these tools.

Transfer learning is the method utilised to transfer old smart home knowledge to a new smart home while taking guidance from the user. Working with the old smart home dataset helps to measure the accuracy of the simulated dataset, and at the same time, gives an opportunity to improve the current knowledge if it is not sufficient to provide the user with the required services. Section 4.4 explains how transfer learning integrates with READY.

This section explains and illustrates how READY uses each of the components in order to provide smart home services to the user. Two users participated voluntarily in this testing process. We have named them user A and user B.

***Scenario:*** *The user wakes up, uses the bathroom, and then goes to the kitchen to make their breakfast. They then eat breakfast, go back to their bedroom, get ready, and go outside.*

The user would expect lights in the bedroom, corridor, bathroom, kitchen, shower, and on the table to come on automatically as needed, as well as a kettle and radio in the kitchen. The user will also expect the switching off all automated devices if they forget to switch them off before leaving the house.

### Survey to understand the daily user activities

Data-driven activity recognition systems predict human behaviour via analysis of the user’s past daily living activity dataset. Unfortunately, a new smart home does not have any records about the user activities or preferences. At this point, we require a technique to enrich the data available to the system. For this reason, we designed a questionnaire [3] to collect daily living information of the user (Fig. 3, step 2). Once the user activity and behavioural data are collected in details, the information will be ready to enter into the system.

#### Collection of activity data

In order to select target activities, a questionnaire must be designed based on the services the user desires. For simplifying, target activities were divided into two categories, namely *simple activity* and *complex activity*. The number and sequence of actions in the *simple activity* category are the same for all users. For example, during the activity of “wake up”, three separate sensors, bed pressure, bedroom motion, and bedroom light detect activity irrespective of the time each activity occurs.

Conversely, the number of actions and their sequence differs for *complex activities*. As an example, making a cup of tea is considered a *complex activity*. There are two ways to prepare tea: some people use milk, some do not, and the sequence of actions in the process can also differ.

In the questionnaire, each user specifies the days that a particular activity occurs, the activity time slots, and the time relation between any two consecutive activities. For example, between 5:00 and 6:00 p.m., the user may enter the house and then go to the kitchen 10 min later to make a coffee.

#### Collection of behaviour data

In this part, our objective is to understand each activity in greater detail to identify factors specific to each user. Data collected will include when the activity occurred, the sequence of activities, their duration and location. Objects used to complete each activity are also important because this information suggests what sensor will be necessary to detect the activity.

#### Survey evaluation with a real scenario

The users (user A and user B) were invited individually for the interview. As previously described, the interview was held face-to-face with a pre-designed set of questions posed to each user. Users were encouraged to explain how they perform various activities naturally.

We only considered monitoring those activities necessary to provide a particular automation facility. According to the above scenario on Sect. 4, the monitoring activities were- wake up, use the bathroom, use shower, make tea, and go outside.

**Table 1:** User weekday activity sequences

User	Time range	Activity and sequences
User A	06:00–07:00 AM	Wake up $\rightarrow$ Use bathroom $\rightarrow$ Make tea $\rightarrow$ Go outside
User B	06:30–08:00 AM	Wake up $\rightarrow$ Use bathroom $\rightarrow$ Use shower $\rightarrow$ Make tea $\rightarrow$ Go outside

**Table 2:** Example of simple and complex activities

Scenario	Simple activity	Complex activity
Morning	Wake up, Use bathroom, Use shower, Go outside	Make tea
Evening	Enter home, Use bathroom, Sleeping	Make tea, Relaxing

**Table 3:** Users’ action sequences for particular activities

Activity name	User	Action involves	New name
Wake up	User A	BedPressure ON $\rightarrow$ BedroomMotion ON $\rightarrow$ BedroomLight ON	N/A
	User B	BedPressure ON $\rightarrow$ BedroomMotion ON $\rightarrow$ BedroomLight ON	N/A
Use bathroom	User A	CorridorMotion ON $\rightarrow$ BathroomDoor OFF $\rightarrow$ BathroomMotion ON $\rightarrow$ BathroomLight ON	N/A
	User B	CorridorMotion ON $\rightarrow$ BathroomDoor OFF $\rightarrow$ BathroomMotion ON $\rightarrow$ BathroomLight ON	N/A
Use shower	User A	CorridorMotion ON $\rightarrow$ ShowerDoor ON $\rightarrow$ ShowerMotion ON $\rightarrow$ ShowerLight ON	N/A
	User B	CorridorMotion ON $\rightarrow$ ShowerDoor ON $\rightarrow$ ShowerMotion ON $\rightarrow$ ShowerLight ON	N/A
Make tea	User A	KitchenDoor ON $\rightarrow$ KitchenMotion ON $\rightarrow$ Kettle ON $\rightarrow$ Cupboard ON $\rightarrow$ Fridge ON	Milk Tea
	User B	KitchenDoor ON $\rightarrow$ KitchenMotion ON $\rightarrow$ Kettle ON $\rightarrow$ Cupboard ON	Black Tea
Go outside	User A	CorridorMotion ON $\rightarrow$ EntranceMotion ON $\rightarrow$ CorridorLight OFF $\rightarrow$ EntranceDoor OFF	N/A
	User B	CorridorMotion ON $\rightarrow$ EntranceMotion ON $\rightarrow$ CorridorLight OFF $\rightarrow$ EntranceDoor OFF	N/A

The time slots and the activity sequences performed within those time slots for both users A and B are in Table 1. Simple and complex activities (Table 2) have been separated and given unique names (labels). For example, we discovered that “Make tea” is the single most complex activity because making tea could be different for different users; some people use milk, some do not, and as a result, the sequences of the action could also be different. We have described the action steps involved in the performance of each of the activities in Table 3.

### User behaviours and smart home simulation

A simulation (Fig. 3, step 4) is designed to establish and acquire user knowledge of a new house, model user behaviour based on user responses, and generate an initial dataset. UbikSim [40] is used to design the simulation.

UBikSim is an open source, Java-based program with a rich library. These features make it easy to integrate with the other components of the proposed system. Originally developed to study complex multi-agent systems (MAS), UbikSim is modified to include new features for this project.

For this project, we utilised UbikSim in two phases. Phase 1: Virtual House Design and phase 2: User Behaviour Design.

#### Virtual house design

In this phase, the developer needs the original floor plans and furniture layout of the new home in order to design the virtual home. UbikSim editor is then used to prepare the virtual floor plan and control different aspects, such as room dimensions and available square footage. Next, furniture, appliances and other home items are added from either UbikSim library or Sweet Smart Home library [1]. UbikSim supports the Sweet Smart Home library, which has an extensive collection of home furniture and appliances. Finally, import required sensors to the smart home from the sensor’s library. All the required sensors are available in the current version of UbikSim, such as motion sensor, door sensor, light sensor, object sensor and pressure sensor. An advantage of UbikSim is that it allows designers to add smart features to any home furniture or appliances easily.

#### User behaviour design

After designing the virtual smart home, the developer needs to decide the context to design the simulation. Here, context means the specific time frame to be simulated such as morning, evening, or afternoon.

In the previous section, the developer allocated a name (Table 3) to each particular activity. So, in this stage, the activity name allocated is assigned as an activity label. Now the virtual home is ready to perform. Before running the simulation, the time and location of the avatar (virtual user) also need to be assigned.

Within UbikSim, an avatar is an interactive object that can move within the virtual smart home and passively or actively interacts with the virtual sensors to represent the behaviour of a real inhabitant. A server records the interaction between the Avatar and the virtual sensors.

This example illustrates how READY engages the user in the simulation process. In the first interview, the developer gathers the required answers needed to simulate user behaviour in the new house. The user becomes familiar with the new home in a virtual environment and examines the simulated behaviour. If the user has agreed that the simulation reflects his or her daily living activity, then the next step in the development process is initiated. Otherwise, the step is repeated.

#### Simulation evaluation with a real scenario

The Smart Spaces Lab of Middlesex University was used to evaluate the scenario as presented in Sect. 4. This Lab contains a living room, a bedroom, a kitchen, a bathroom, a shower room and a corridor space.

**Fig. 4:**
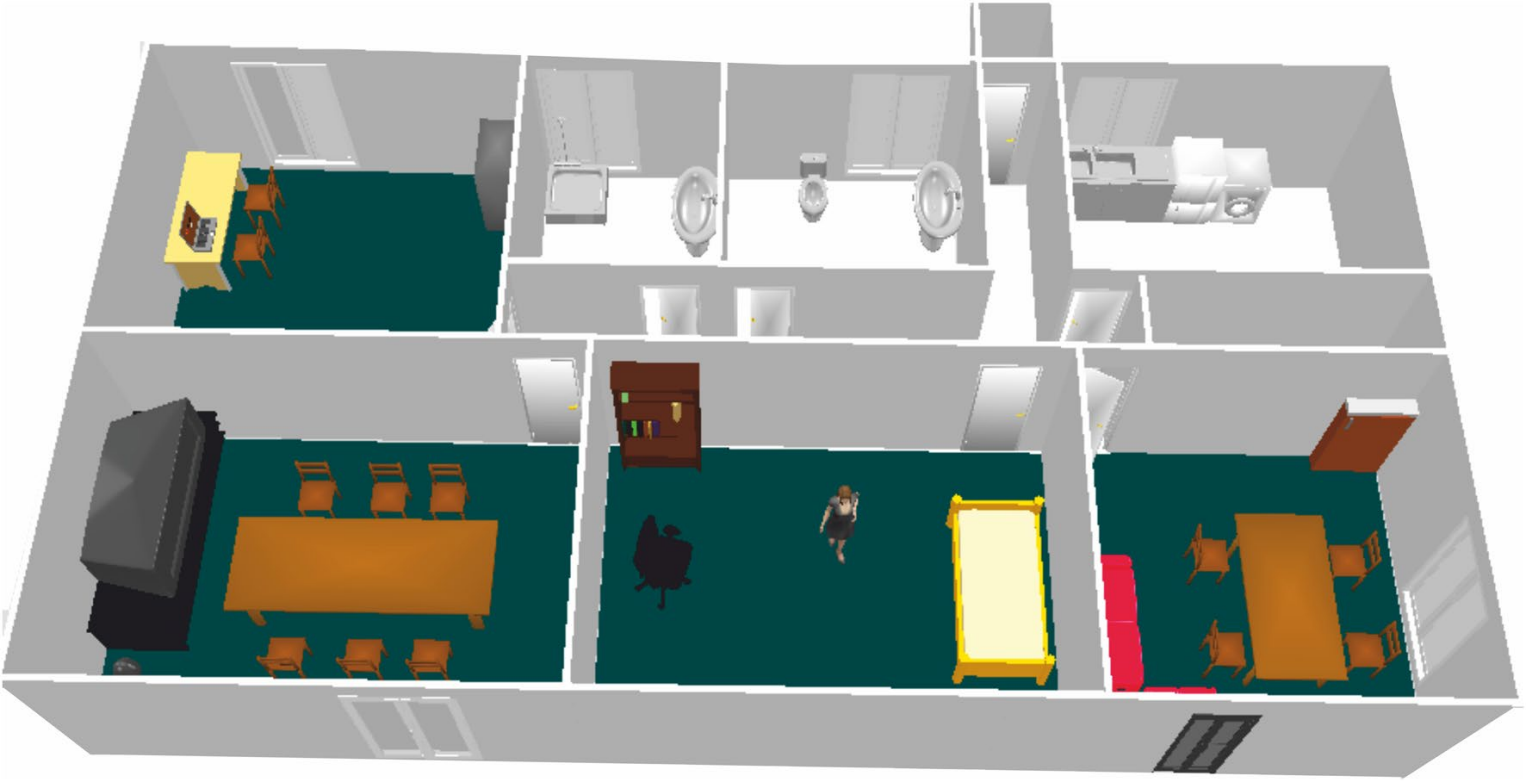
Simulation of the Middlesex University smart space lab

To facilitate the simulation, we created a virtualised replica of the physical Lab environment using UbikSim editor. We paid particular attention to ensure that the virtual environment looked precisely like the Lab environment and that none of the independent sensors and house appliances embedded with a sensor should be excluded. The results of this simulation are shown in Fig. 4.

In a second interview, we examined and validated the simulation of expected user behaviour based on the information provided in Tables 1, 2 and 3. In addition, the simulation should be modified to include any newly discovered information and any information that had been overlooked or inadvertently omitted from prior consideration.

Table 4 shows feedback received from users A and B. This feedback contains vital information for the further development process. For example, user A corrected that he usually sits on the bed 5–10 min, and he also does not take a shower in the morning. According to user B, his first activity after waking up is turning on the kettle in the kitchen, creating a change in the sequence of activities. Hence, we shall modify our simulation accordingly and, finally, generate the dataset.

**Table 4:** The feedback received from the users

Activity name	User	Feedback	Sequence #
Wake up	User A	After waking up user wait 5–10 min on the bed	1
	User B	Accepted, no feedback	1
Use bathroom	User A	Accepted, no feedback	2
	User B	The user goes to the kitchen before the bathroom to put the kettle on	3
Use shower	User A	N/A	N/A
	User B	Accepted, no feedback	4
Make tea	User A	Milk tea	3
	User B	Black tea	2
Go outside	User A	Accepted, no feedback	4
	User B	Accepted, no feedback	5

### User activity recognition

This section describes how we use activity recognition, translator, and reasoner tools to provide the user with smart home services. We offer a thorough explanation of LFPUBS, LFPUBS2M and MReasoner in the following sections.

The aim of providing the user with smart home automation services includes making their life more comfortable, safer and more energy-efficient. Human beings exhibit behaviours based on their habits. We propose that a user’s past and present behaviours are also indicative of their future behaviour patterns.

The project used LFPUBS to identify behaviour patterns from within the users daily activity dataset. LFPUBS software consists of a three-layer architecture, with each layer playing a vital role in data representation. The data representation gives extra advantage to the developer to visualise the sequential transformation of the data. Moreover, this representation feature of the LFPUBS software convinced us that it was the most appropriate software for this project.

LFPUBS also provides user-centric design, consistent with the overall goal of this project, in which we collect user data and act intelligently for the user. The system LFPUBS is based on three-layered architecture.

The *transformation layer* transforms the raw data collected from the sensors. It processes the dataset so that data is represented as an action string with a temporal ordering without any particular structure. Introduced data is collected and split into sequences to determine its meaning; the transformation of this data into simple actions, which are then combined, provides further understanding of its significance.

The *Learning Layer* transforms meaningful information passed to it from the Transformation layer into knowledge and is the core of the system. In this way, the learning layer is kept free from external influence.

The Learning layer uses two different approaches to create knowledge from the information passed to it, namely the Action Map. This approach discovers user behaviour patterns and represents them in a comprehensible way, and a pairwise approach focuses on discovering pairwise relations between actions of the user.

The Learning layer consists of two separates but fully integrated modules: a representation module and a discovery module.

Representation module: This module uses a language that represents patterns based on Event-Condition-Action (ECA) rules. It also provides a standard way of describing patterns, making sure that those patterns are specified and use other technologies to check their integrity. Like ECA rules, LFPUBS language ties up two actions (ON and THEN clauses) and one condition (IF clause). It also describes the time relation between both actions.

Discovery Module: This module uses a proprietary learning algorithm to discover the frequent behaviours of the user. The algorithm processes in four phases. First, it identifies the frequent sets of actions, then the topology, followed by the quantitative time relations and conditions.

The *Application layer* shows or uses the knowledge generated by the learning layer. Different human-computer interfaces (HCI) can then be developed for a specific learning process.

#### LFPUBS2M

After detecting the user behaviour, we require a reasoning system that acts in real-time and provides automation services based on acquired knowledge. A reasoning system called MReasoner, used for this project, is explained in the following section.

MReasoner and LFPUBS have different functionalities both in format and content. Therefore, a coupled active system translation tool developed and named LFPUBS2M, was used to enable LFPUBS to talk to MReasoner.

#### MReasoner

MReasoner is a system designed in “M” language [29]. It defines the context of interest based on the natural characteristics of reactive intelligent environments and can track certain environmental conditions and act upon those. Moreover, MReasoner implements the forwards reasoning algorithm explained in [29]. Furthermore, the database is used to communicate between the MReasoner and the real world.

MReasoner uses an inference engine to perform reasoning. It then repeats the application until it reaches the goal. The inference engine starts with the available data and inference rules. Then, it utilises a forwards reasoning search until it finds that the antecedent (if clause) is known to be true. When found, the engine can conclude, or infer, the consequent (Then clause), resulting in latest information to the data. Inference engines will iterate through this process until the achievement of the goal.

In the READY method, data generated from UbikSim is transferred to LFPUBS (Fig. 3, step 9) to enable the extraction of behavioural and activity patterns. These patterns, once converted into M language rules by LFPUBS2M (Fig. 3, step 10), are then installed within an instance of MReasoner (Fig. 3, step 11) within a new house. User feedback will ensure that MReasoner has automated the house in a desirable time. If not, then the rules are modified according to the system feedback (Fig. 3, step 15 & 16).

#### System evaluation with a real scenario

The evaluation process described in Sect. 4.2.3 resulted in the generation of a simulation dataset for user A and user B. This section explains the details of that evaluation and explains how LFPUBS and LFPUBS2M process the simulation data to generate M rules, thereby providing automation services to user A and user B.

Before generating the initial simulation dataset, the developer needs to understand how LFPUBS identifies patterns or risk the generation of invalid patterns. The sequences of activities performed by a user (avatar) within the simulation are initially defined within LFPUBS by the developer. LFPUBS discovers normal relations from these sequences. So, the developer should be aware of this behaviour before generating a simulation data set.

Raw simulation data requires conversion into a particular format for use within the LFPUBS system. Section 4.3 describes the internal architecture of the LFPUBS system that has several options that allow the developer to process the dataset and extract the prospective knowledge from the data.

LFPUBS2M is a translator that works as a bridge between LFPUBS and M Reasoner, enabling the conversion of LFPUBS generated patterns into M rules.

**Table 5:** The results received after evaluating the scenario

Activity name	User	Expected services	Service received
Wake up	User A	The bedroom light on when user wake up	No light on
	User B	The bedroom light on when user wake up	Light on
Use bathroom	User A	Bathroom light on when user wants to use the bathroom	Light on
	User B	Bathroom light on when user wants to use the bathroom	Light on
Use shower	User A	Shower light on when user go for a shower	N/A
	User B	Shower light on when user go for a shower	Light on
Make tea	User A	Kettle on when the user decides to make tea	Delay to trigger the kettle
	User B	Kettle on when the user decides to make tea	Kettle on
Go outside	User A	All house light turn off when user left the house	Delay to turn off all the light
	User B	All house light turn off when user left the house	Light off

In Table 5, the first column lists the activity name we detect to provide the expected services. In the test results evaluated for user A, the bedroom light does not turn on when the user A wakes up. Furthermore, we can see that both the ‘Make tea’ and ‘Go outside’ activity triggering times were late. Careful analysis revealed that the bedroom light did not turn on because of a faulty PIR (passive infrared) sensor and MReasoner had failed to make the automation delay.

**Fig. 5:**
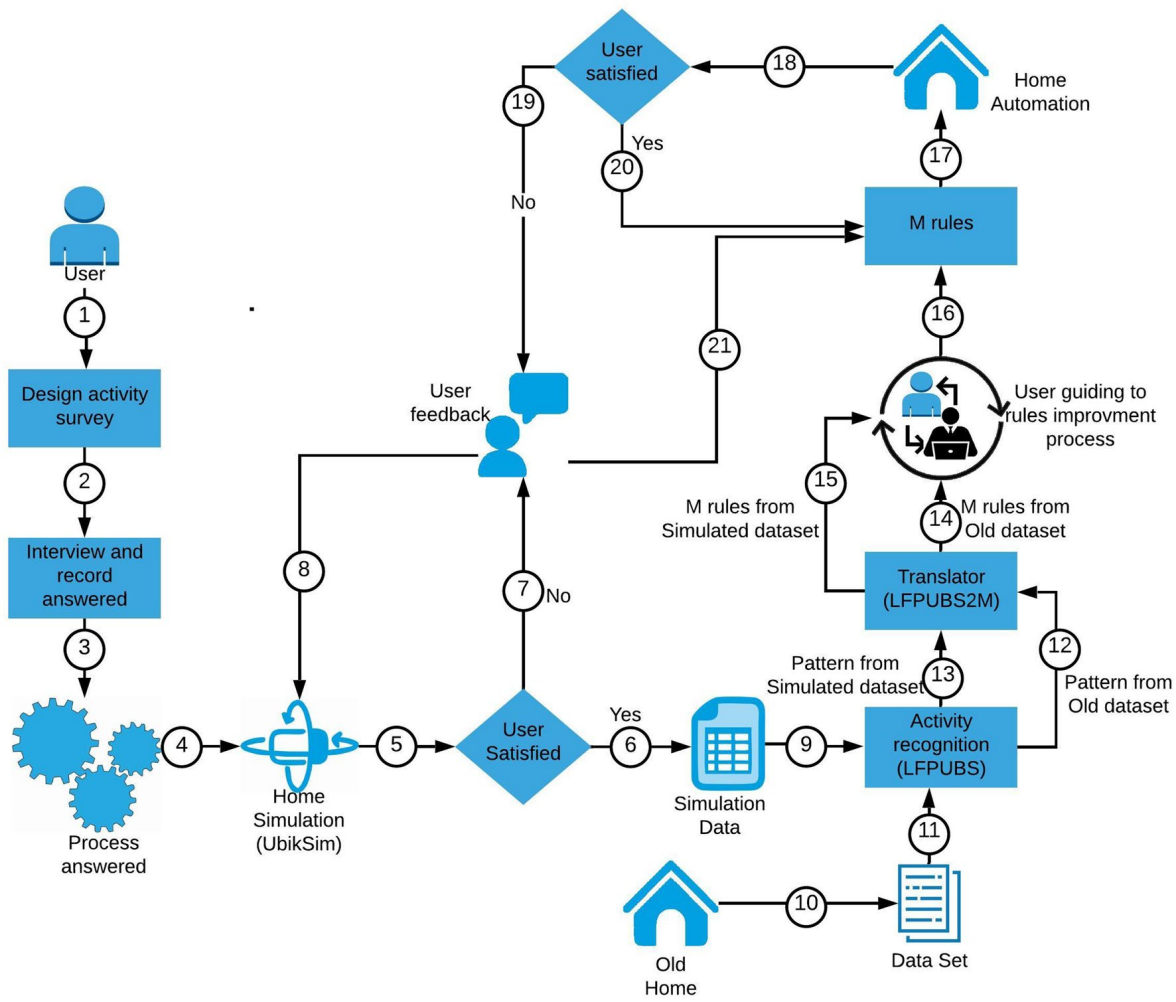
System Architecture: READY system extended to UTL

### User-guided transfer learning (UTL)

The objective of the system is to provide automation services to the user as soon as the user starts living in a smart home. The MReasoner computational model ensures that automation happens through the execution of “M” rules.

As shown in Sect. 4.3, the system can initially provide a level of automation service with “M” rules selected based on prior experience and user preferences from a simulation dataset. However, a significant question now needs to be addressed. How accurate is the set of “M” rules in use, and can these rules be further modified to more efficiently satisfy the users’ needs for smart home automation?

With transfer learning, a system can leverage experience from a previous task to improve the performance of the new task [23]. To answer our question, we propose a User-guided Transfer Learning (UTL) approach, where new house knowledge is improved by old house knowledge to increase overall automation functionality and effectiveness. We also utilise user knowledge and feedback to ensure that improvements are appropriate for the user.

It is important to note houses with different physical or technological configurations pose a challenge. The adaptation we do is of the same person so habits are still meaningful. Naturally the closer the resemblance of the new place to the previous one the smoother the adaptation is and vice versa.

Although we focused on a same user system adaptation, in principle our approach can also be used with data and rules generated from a different user. However, the number of iterations required to converge into satisfaction will be proportional to how similar the habits and technology are in the source data and rules.

Figure 5 shows an overview of the process. First, the old smart home data should be collected and processed. Data is then entered directly into the LFPUBS. LFPUBS extracts the most frequently observed patterns of user behaviour from the dataset. This pattern data is then passed to the LFPUBS2M translator to generate the relevant “M” rules (Table 6).

Similarly, simulation data is also entered into the LFPUBS system to find the most frequent patterns that emerge from the simulation dataset. These patterns are also passed through the LFPUBS2M for translation into “M” rules.

Two sets of rules are ready to be considered; one set received from the simulation dataset (Sect. 4.3) and another from the old smart home dataset. The developer analyses the old smart home dataset rules to the current set of rules one by one (details in Table 7). If the developer identifies a benefit, and if the user approves of the suggestion, the improvements will be implemented by the M system. User feedback can be sought and incorporated into future sets of upgraded rules as often as necessary.

#### UTL evaluation with a real scenario

This section recalls the scenario from Sect. 4 for evaluation using the UTL method. Section 4.3.3 shows that the system is capable of providing the requisite level of smart home services to user A and user B.

The old smart home dataset for user A and user B is vital in continuing the evaluation process. In reality, however, for the experiment, it is often either impractical or impossible to find the old smart home data for user A and user B, which creates a data unavailability problem.

To overcome the old data unavailability problem, research participants performed the above scenario in the morning, in the Middlesex University Smart space Laboratory for 4 weeks and saved the resulting dataset to the server. Patterns were then generated from this dataset by LFPUBS (Fig. 5, step 12), and LFPUBS2M was used to translate these patterns to MReasoner rules (Fig. 5, step 14).

As described previously, one of the reasons that LFPUBS and MReasoner were selected is that the outputs produced are human-understandable. This feature critically supports the developer throughout the system development process.

To fully understand the proposed approach, we encourage the reader to focus on the “User guiding rules for improvement process” section in Fig. 5, where the developer and user sit together with two sets of rules. Developers consider only those rules which do not exist in a simulated set of rules. Considering that the user had minimal knowledge about the technology, the developer must then explain the purpose of each rule in user-friendly language and question the need for any new rules.

In Table 7 (in bold), the developer found a new set of rules showing that user A takes a bath on Friday morning. So, at this point, the developer will ask user A if they take a bath every Friday morning. If the answer is yes, then a new rule would be added to the system. If not, then a new rule would be unnecessary. However, user A may now decide that they want to add this facility as an automation service during the evaluation process. Alternatively, they could have omitted to specify this service in the first phase. Irrespective, the addition of a new automation rule by the developer is straightforward.

For user B, the current rules state that they go outside every weekday at 5 AM. However, user B confirmed that this rule would not be necessary at the new house. So, the developer does not add this rule into the system.

## System testing and evaluation

The most valid and reliable method of testing within the IE area is to test a smart home automation solution in a real environment and observe user interactions over an extended period of time. Specifically, it is the interaction between the user and the system that makes it possible to assess whether the system indeed provides the promised services.

This paper has discussed various approaches to both testing and evaluating automated smart homes. This section delves into the experimental results gathered from a comprehensive test of the READY method. This system testing and validation reflects a more developed and refined version of the preliminary work discussed at the SGAI International Conference [6].

Validation is a challenging endeavour because home automation systems are complex collections of sensors, networks, databases, humans, software, infrastructure, and environments. If any one of these elements fails, then the system as a whole will not produce the correct results. For example, in Sect. 4.3.3, the test failed due to a faulty PIR sensor. For this reason, when testing a system of this level of complexity, it is imperative to consider each and every component of the system so that any system failures can be traced back to their source.

To address this need, [14] introduced the COATI method. The COATI method considers a smart home to be a complete system with the resources needed to deliver services in a specific context. These resources are referred to as *enablers*.

The approach also proposes a table (called *check table*) that highlights the minimum system configuration required for a context-specific solution to work in order to avoid a situation where an element may fail. The COATI method was adopted for system testing of the current project. Section 5.1.2 illustrates in depth how COATI assists in testing the system. To test the prototype, we examined system performance in two scenarios, described below. Although the scenario in the article is rather simple, this methodology can be applied to more complex scenarios and also to more important contexts (for example having an impact on well-being and health).

*Scenario (Morning)*: The user wakes up, uses the bathroom, goes to the kitchen to make breakfast, eats breakfast, goes back to the bedroom, gets ready, and goes outside.

In this scenario, the user would expect several devices (e.g., lights, tea kettle) in the kitchen, dining room, bedroom, corridor, bathroom and shower to automatically switch on and off.

*Scenario (Evening)*: The user comes back from the office, changes clothes, uses the bathroom, goes to the sitting room, reads the newspaper, goes to the kitchen, makes dinner, eats dinner, and goes to bed.

Again, the user would expect all automated devices to turn on when appropriate and switch off by the time they go to bed.

These scenarios were used to test the ability of the newly developed READY approach, in combination with user-guided transfer learning, to mitigate the cold start problem. Thus, a two-pronged testing strategy was undertaken, with the first focused on testing the READY method and the second focused on testing the UTL method.

### Solving the cold start problem using the READY approach

**Fig. 6:**
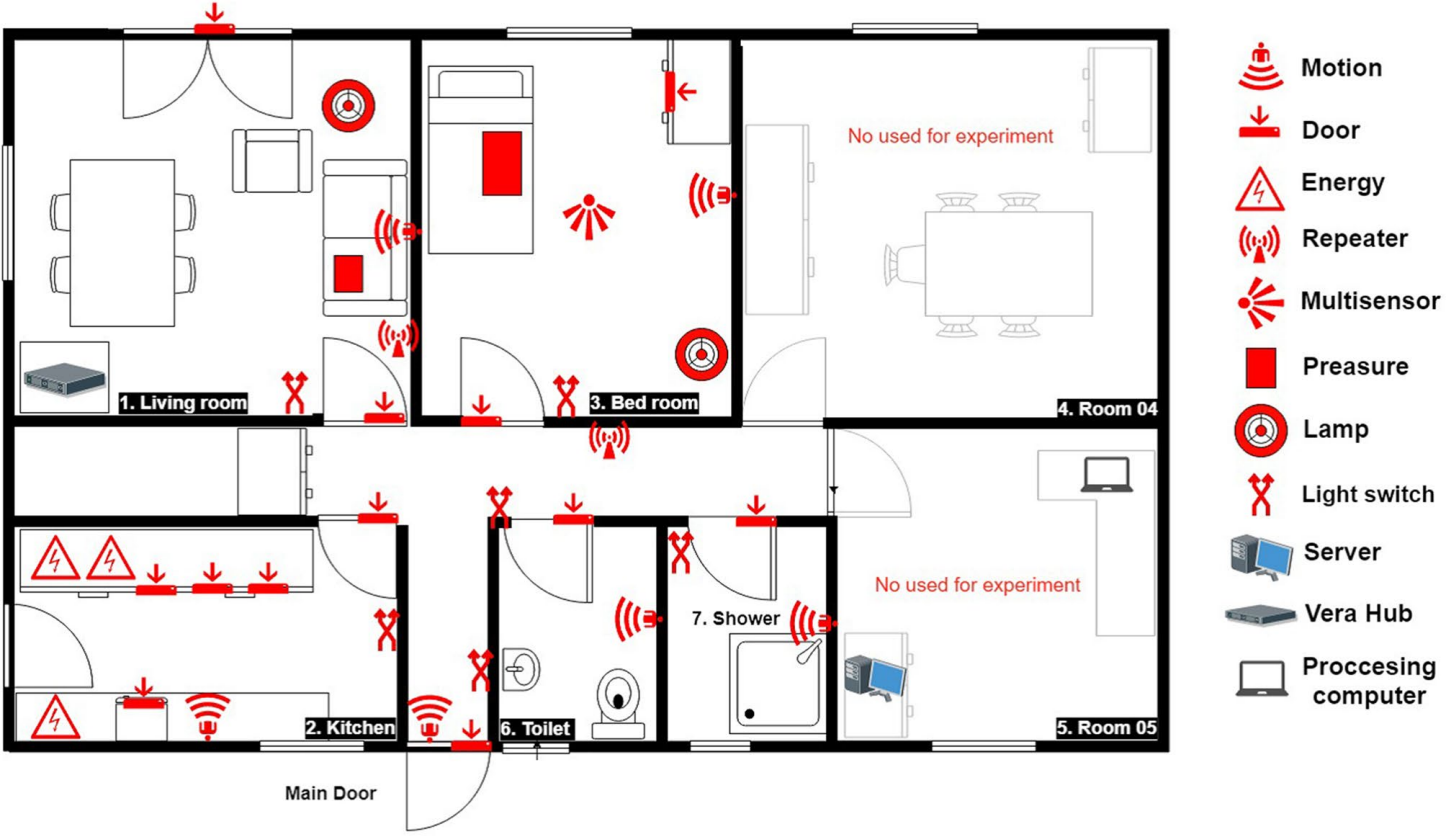
A map of the lab including sensor hardware

The READY approach was tested on the above two scenarios with a set of experimental tools that aimed to determine whether the system could satisfy the specified requirements. Although there are many ways to test smart home automation systems, the present study adopted a System Action approach (see LFPUBS Sect. 4.3) to observe user interaction (Fig. 6).

**Fig. 7:**
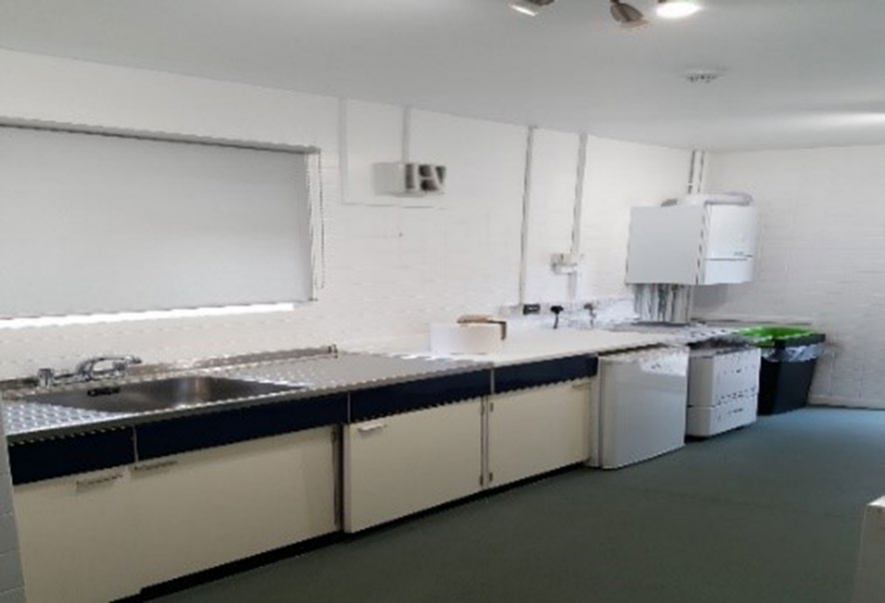
Kitchen of the Smart Spaces Lab

**Fig. 8:**
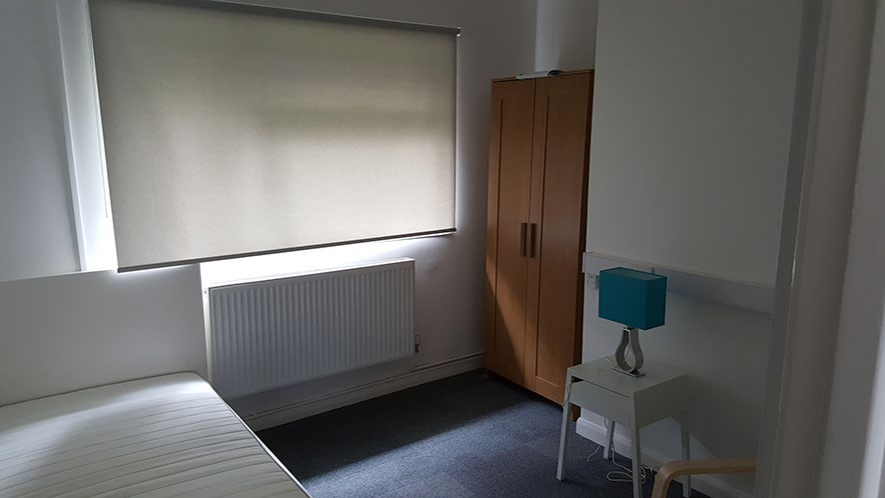
Bedroom of the Smart Spaces Lab

The assessment was conducted within the Smart Spaces Lab at Hendon Campus in Middlesex University. The lab environment consisted of a house with a living room, (Fig. 7), bathroom, shower, large bedroom (Fig. 8) and office. Sensors were installed in the house shown in Fig. 6.

**Table 6:** Name, type and quantity of sensors installed in the smart space

Sensor name	Sensor type	Sensor quantity
Motion sensors	PIR	6
Door sensors	Door	11
Object sensors	Power	3
Light sensors	Light	6

**Table 7:** The results received after evaluating the UTL

Activity name	User	Expected services	Service received from simulated dataset	Service offered by previous home dataset
Wake up	User A	The bedroom light on when user wakes up	No light on	Light on
	User B	The bedroom light on when user wakes up	Light on	Light on
Use bathroom	User A	Bathroom light on when user wants to use the bathroom	Light on	Light on
	User B	Bathroom light on when user wants to use the bathroom	Light on	Light on
Use shower	User A	Shower light on when user goes for a shower	N/A	**Turn on light on Friday**
	User B	Shower light on when user goes for a shower	Light on	Light on
Make tea	User A	Kettle on when the user decides to make tea	Delay to trigger the kettle	Kettle on without delay
	User B	Kettle on when the user decides to make tea	Kettle on	Kettle on
Go outside	User A	All house light turn off when user leaves the house	Delay to turn off all the light	Light off without delay
	User B	All house light turn off when user leaves the house	Light off	**Turn house light off as user go outside morning at 5 a.m as well**

New service detected from the old smart home dataset in bold

Specifically, PIR motion sensors were installed in the kitchen, corridor, entrance, living room, bedroom, bathroom and shower to detect user movement. Additionally, door sensors were installed in every door of the house, including the doors of the kitchen cupboards and refrigerator, to detect when doors were opened or closed. These sensors were used as indicators of user interactions with the objects. For instance, BedroomLamp referred to the status of the lamp installed in the bedroom. Similarly, Kettle indicated the status of the tea kettle in the kitchen.

Five individuals were invited to take part in the system validation process, which was performed in accordance with Middlesex University data protection regulations. Prior to testing, each individual was provided with a basic knowledge about the smart home and the validation process.

Given that each automation solution is unique to a single user, only one participant could test the system at a time. To test the scenarios described above, participants were invited to the lab for both morning and evening sessions.

#### First session

In the first session, participants responded to a questionnaire (Sect. 4.1) asking for information on how they perform morning and evening daily living activities. Although participants likely perform a variety of activities, only eight activities were selected for the purposes of testing. These activities exemplified tasks or behaviours that were presumed to be applicable to a majority of prospective users (see Table 8). Participant responses were recorded and organized (Table 9) to make the simulation design more straightforward.

Table 9 shows a simulation of participant behaviour in a virtual smart home. More details on the virtual smart home development and behaviour simulation process are available in Sect. 4.2.

**Table 8:** The activities considered for the validation process

Scenario	Simple activity	Complex activity
Morning	Wake up, Use bathroom, Use shower, Go outside	Make tea
Evening	Enter home, Use bathroom, Sleeping	Make tea, Relaxing

**Table 9:** Participants answers organized to design the simulation

User	Time range	Scenario	Activities and sequences
User A	08:30–09:30 AM	Morning	Wake up $\rightarrow$ Use bathroom $\rightarrow$ Make tea $\rightarrow$ Go outside
	07:00–10:00PM	Evening	Enter home $\rightarrow$ Use bathroom $\rightarrow$ Relaxing $\rightarrow$ Make tea $\rightarrow$ Sleeping
User B	06:00–07:00 AM	Morning	Wake up $\rightarrow$ Use bathroom $\rightarrow$ Make tea $\rightarrow$ Use shower $\rightarrow$ Go outside
	08.00–10.00PM	Evening	Enter home $\rightarrow$ Make tea$\rightarrow$ Relaxing$\rightarrow$ Make tea $\rightarrow$ Sleeping
User C	08:30–09:00 AM	Morning	Wake up $\rightarrow$ Use bathroom $\rightarrow$ Use shower $\rightarrow$ Make tea $\rightarrow$ Go outside
	08:00–10:00PM	Evening	Enter home $\rightarrow$Make tea $\rightarrow$Sleeping
User D	06:00–07:00 AM	Morning	Wake up $\rightarrow$ Use bathroom $\rightarrow$ Use shower $\rightarrow$ Make tea $\rightarrow$ Go outside
	08:00–11:00PM	Evening	Enter home $\rightarrow$ Use bathroom $\rightarrow$ Make tea $\rightarrow$ Sleeping
User E	08:30–09:00 AM	Morning	Wake up $\rightarrow$ Use bathroom $\rightarrow$ Make tea $\rightarrow$ Use Shower $\rightarrow$ Go Outside
	08:00–10:00PM	Evening	Enter home $\rightarrow$ Make tea $\rightarrow$ Relaxing $\rightarrow$ Sleeping

#### Second session

In the second session, user behaviour was simulated (see Table 9). Then participants were invited to examine the simulation and record their feedback on how well it captured the way in which they would carry out those eight activities. Table 13 summarises the feedback obtained. For instance, as can be seen in Table 13, User A usually rested in bed for a period of 5–10 min after awakening. They left the house before 7:30 am and went to bed before 10 pm. User B accepted the simulation without any feedback. User C requested changing the timing and sequence of actions for making tea. The timing around returning home was also adjusted for User C based on their typical return time of about 8 pm. User D accepted the simulation without any complaint, though he did seek prior assurance that the simulation would correctly simulate the actions and timing associated with making tea and relaxing. Finally, User E accepted the simulation without any feedback.

As stated in Sect. 4.2, the avatar carried out the activities in a predefined way. These passive interactions between the avatar and the virtual sensors were saved in a server for later use by LFPUBS (see Sect. 4.3). In the following section, we explain the parameters used for this project.The performance of the avatar’s predefined activities made it possible to generate labelled data. Therefore, LFPUBS knew in advance what knowledge it would uncover. Table 9 displays the sequence of activities carried out by the avatar.

LFPUBS, an activity recognition system, operates by identifying frequent patterns of user behaviour. More specifically, the LFPUBS system develops its topology by considering discovered repetitive actions. According to [16] - developed topologies cannot guarantee the inclusion of all frequent relationships because frequent relations are often discovered without first establishing a minimum support. Further, a relation may be classified as frequent so long as it reaches the pre-set confidence level, even if it occurs infrequently.

**Table 10:** LFPUBS parameters values to process the simulation dataset

Activity name	Confidence level (%)	Automation device
Wake up	80	BedroomLight
Use bathroom	85	BathroomLight
Use shower	90	ShowerLight
Make tea	90	Kettle
Go outside	90	CorridorLight
Enter home	85	CorridorLight
Sleeping	80	BedroomLight
Relaxing	90	TableLamp

In contrast, frequent sets use a minimum confidence level that also functions as the minimum support level, meaning an action must fulfil the requirement of including minimum levels in a frequent set. This, in essence, is why frequent relations are excluded from topologies. The basic algorithm is used to calculate Time Relations. The number of Conditions was low due to the small number of context sensors used for the experiment. The purpose of automation is to find the most reliable automation path. Table 10 defines the parameters considered to achieve the most reliable path.

The main purpose of this session was to install the system with the rules received from the previous section in the Smart Space Lab, invite participants to perform the scenarios naturally, and observe that automation.

Before executing the tests, all sensors and actuators were inspected to ensure that they performed correctly. They were saved in the system database so they could be connected back to the device number of the log reader. Section 4.3 outlines the steps by which MReasoner executes the rules.

As mentioned at the beginning of this section, the COATI approach influenced the creation of a customised check table. Using this table, it was possible to ensure that all system components were working correctly, and to proceed by turning the focus to rule execution. Table 14 demonstrates an example for User A illustrating how the table was used to check each component of the system. The first column shows the parameters that were considered; the second column catalogs enablers, which are specific contexts that require a certain number of resources from the infrastructure for the context to happen [14]; the third column shows the initial values of the enablers prior to testing; finally, the fourth column displays the number of tests needed to be conducted for the focal context. There is no particular limit to the number of tests required to determine a successful result for any of the experiments. Thus, the process continues until the test is successful. Table 14 shows the example for User A, for whom the first test failed due to a faulty kitchen movement sensor. After repairing the sensor, however, the test was successful.

Pertaining to user tests of the system, the five participants were invited one at a time into the lab. Before testing, the table was checked to ensure that all Enablers were recording the initial value. Then, participants performed their daily activities in any way they so choose. When the sequence of activities was complete, the MReasoner log was checked to identify and repeat any failed tests. This testing process produced ten total tables for the five users, which can be found online in [4].

**Fig. 9:**
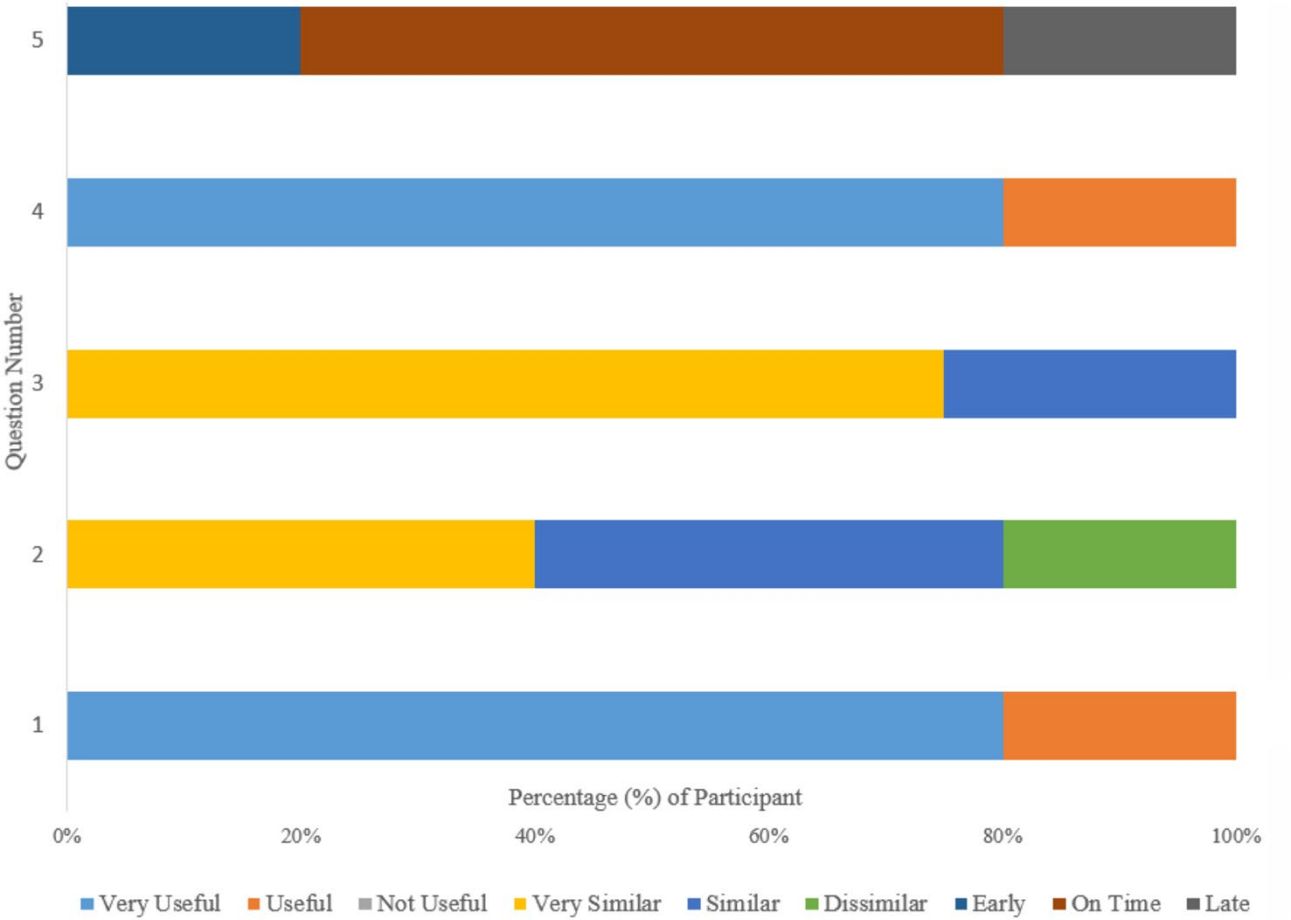
Users’ responses based on questions in Table 11

After finishing this part of the assessment, we asked the participants six questions (Table 11) to measure their acceptance and satisfaction with the new system. The answers received from the users were analysed against a three-point Likert scale (Fig. 9). After finishing this testing, participants were asked to answer six questions (Table 11) to measure their acceptance of and satisfaction with the new system on a three-point Likert scale (Fig. 9)

**Table 11:** Measuring the acceptance of the system and user satisfaction

Number	System acceptance and user satisfaction question
1	How useful is it that the smart home provides services from day one?
2	How similar were the simulated and real smart home solution?
3	How close was the simulated behaviour to the answers you provided?
4	How useful was the simulation in adjusting to the real house?
5	How well did the house provide its automation services?
6	What improvements would you make to the system?

Eighty percent of users responded that smart home automation was beneficial from day one. Further, the availability of the smart home simulation had helped the user adapt to their new home. Interestingly, users did not find substantial similarities between the simulated and real homes. However, they did observe that smart homes provided them with an idea of how the actual house would perform. A majority of users agreed that there were significant similarities between actual and expected simulation behaviours. In general, users were satisfied with the time taken for home automation performance. Sixty percent of users agreed that automation actions occurred within a reasonable time.

### Enhance the understanding of the new home using the UTL approach

This part of the testing is supplementary to the previous part of the testing. Three of the original five participants participated in this part of the validation process, namely users A, B, and C.

In Sect. 4.4.1, we discussed how we avoided the unavailability of the old data set by utilizing the dataset created by each user as they performed daily activities over 4 weeks in the Smart Space Lab. Data saved to the lab server was processed and used as an input for LFPUBS. The LFPUBS parameter was constant for all data sets in the first part of the validation process because when the simulator generated the data, we assured the data accuracy. On the other hand, LFPUBS parameters changed to process different sets of real smart home data. Table 12 shows the LFPUBS parameters provided the most reliable automation path. The output result was used for LFPUBS2M to translate into M rules. The rules are available in [5] (Tables 13, 14).

**Table 12:** LFPUBS parameters values to process the real dataset

Activities	User A (%)	User B (%)	User C (%)	Automation device
Wake up	70	70	75	BedroomLight
Use bathroom	95	70	80	BathroomLight
Use shower	90	70	95	howerLight
Make tea	90	85	90	Kettle
Go outside	80	85	80	CorridorLight
Enter home	90	90	80	CorridorLight
Sleeping	70	70	90	BedroomLight
Relaxing	70	85	90	TableLamp

**Table 13:** The feedback received from the users

Activity Name	User A	User B	User C	User D	User E
Wake up	After waking up user wait 5–10 min on the bed	Accepted, no feedback	Accepted, no feedback	Accepted, no feedback	Accepted, no feedback
Use bathroom	Accept the simulation	Accepted	Accepted	Accepted	The sequence is not right, user drinks tea before goes to bathroom
Use shower	Not applicable	Accepted	Accepted	Accepted	Accepted but user has a concern about the duration of the activity use shower
Make tea	Accepted	Black Tea	Correct the sequence	Milk tea	Accepted
Go outside	User left the house before 7:30 am	Accepted	Accepted	Accepted	Accepted
Enter home	Accepted	Accepted	User enter home after 8 PM at Monday	Accepted	Accepted
Sleeping	User sleep around 10 PM	Accepted	User does have any particular time to goes for sleeping	Accepted	Accepted
Relaxing	Relax in Bedroom	Relax on the setting room	sleeping	Relax on sofa	Relax on setting room

**Table 14:** Participants answers organized to design the simulation

Morning scenario	Enablers	Assumptions initial values	Test 1	Test 2
Context description			Facilitate the user daily morning activities to automate the home equipment	
Expected outcome(s)			The lights in the bedroom, corridor, bathroom, kitchen, shower, and on the table switch on automatically when required, as well as the kettle in the kitchen. All automated devices will be switched off if the user forgets to switch them off before leaving the house	
Real outcome(s)			The kettle does not turn on	The user received the required services
Sensors	EntranceMotion	EntranceMotion=0	EntranceMotion=1	EntranceMotion=1
	CorridorMotion	CorridorMotion=0	CorridorMotion=1	CorridorMotion=1
	BedroomMotion	BedroomMotion=0	BedroomMotion=1	BedroomMotion=1
	KitchenMotion	KitchenMotion=0	KitchenMotion=0	KitchenMotion=1
	BathroomMotion	BathroomMotion=0	BathroomMotion=1	BathroomMotion=1
	ShowerMotion	ShowerMotion=0	ShowerMotion=1	ShowerMotion=1
	SettingMotion	Not applicable for this context	Not applicable for this context	Not applicable for this context
	EntranceDoor	EntranceDoor=0	EntranceDoor=1	EntranceDoor=1
	FrezzerDoor	FrezzerDoor=0	FrezzerDoor=1	FrezzerDoor=1
	Cupboard	Cupboard=0	Cupboard=1	Cupboard=1
	Kettle	Kettle=0	Kettle=0	Kettle=1
	BathroomDoor	BathroomDoor=0	BathroomDoor=1	BathroomDoor=1
	SmallPaddle	Not applicable for this context	Not applicable for this context	Not applicable for this context
	BigPaddle	BigPaddle=1	BigPaddle=1	BigPaddle=1
	TableLamp	Not applicable for this context	Not applicable for this context	Not applicable for this context
Network	Z-wave (Vera hub)	Vera has a connection with sensors involved	There is a connection with all the sensors and update their value	There is a right connection
Database	Monitoring Database	Added the sensors and actuators to the database	Database updated, the sensors and actuators status value has been changed	Database updated, the sensors and actuators status value has been changed
Reasoner	Connection with sensors and server	The tools connect with Vera and MReasoner	The info from Vera is updating in the tool. There is a connection with the server.	The info from Vera is updating in the tool. There is a connection with the server
User	user A	user A	user A	user A

Now that we have two sets of rules, we can borrow the simulation dataset from the previous validation section and obtain another set of rules from the real dataset. We only consider those rules which are not available in the simulated set of rules.

After analysing the two sets of rules, we separated those rules that only exist in the rules of the real dataset to find out if the rules can provide new services. We invited each participant to register their interest in the new services. If a participant expressed interest, we created a new set of rules. Table 15 shows the new services proposed for each user. The modification rules are available in [5]. The new services are implemented to the home with considering the user acceptance.

**Table 15:** New service detected from the old smart home dataset

Participants	Services detect from real dataset
User A	User enter home at 4 PM on Friday
User B	No new service detected
User C	The system detect the bed room light off between 9–10 PM

### Discussion of the testing outcome

Smart home adaptation has been a popular research domain for a number of years. Over this time, researchers have developed and used a wide range of approaches to improve the smart home adaptation process. Our system development approach engages the user in a way that makes it appear that the system subsequently developed was based upon a blueprint of the user requirements, empowering the smart home to provide specified services to its resident as soon as it is installed. Further, the user is so closely involved in the system development process that they are fully aware of the system’s operation before they start living in the house.

In light of the results outlined in Sect. 5.1, it is reasonable to conclude that the system can provide the smart home services to the user as soon as occupancy begins. A practical and satisfactorily performing home automation system increases user peace of mind and will increase the popularity of the smart home.

Researchers have been using simulation as a tool for the development of smart home services for more than a decade. Its use, however, has been limited to experimental purposes as it does not ordinarily come packaged as an integrated component of a home automation system.

The READY approach uses simulation for core system development. As an interface, simulation can use both observed and survey response behaviour and effectively transfer this behaviour to the system. In this research, we sought to highlight the effectiveness of various tools operating together to solve a specified problem rather than extol the benefits of specific tools, and it was for this reason, that we did not focus on developing sophisticated simulation tools. Ultimately, we discovered that users identified substantial differences between the simulation generated by UbikSim and the real home.

We did establish that simulation was sufficient to provide each user with a basic idea about the performance of automation services within their future house. Further, we found that a simulated solution was more effective in eliciting likely user behaviour in the real house and generating valuable datasets for pattern recognition and analysis.

LFPUBS is an ideal tool for identifying frequent and recurring patterns within datasets. The greater the dataset available for analysis, the better it was observed to work. LFPUBS finds it much easier to identify patterns within datasets generated by simulation than real datasets. This may be due to the absence of sensor-generated errors, such as a missed or incorrectly turned on or off sensor. We found that sufficient data to develop a system solution was generated by simulation, thereby meeting the experiment requirements.

Four weeks of daily activity data generated by user interaction with the home using the UTL approach was time-consuming but did reveal several exciting patterns that the simulation was unable to identify. These patterns became new automation services that the users tested during the next part of the testing (Table 15).

A review of user feedback (Fig. 9) confirmed that the system was able to provide the required automation services successfully. However, two users observed that kettle automation did not happened as expected when making tea. Inspection of the relevant sensors revealed that the Kitchen movement sensor reset time was more than 20 sec. This incorrect parameter setting thus resulted in an unexpected delay within the automation process.

There were a number of challenges researchers encountered. The study was limited to a small group of user participants. The UbikSim and LFPUBS tools had to be extensively customised to ensure that they would work seamlessly together for this project. Each generated smart home dataset was unique to a single occupant. Constant monitoring of the system was necessary to ensure that the data set used to provide automation services was the correct one for the specific user.

### Evaluation

As discussed in earlier chapters, U-CIEDP is a system development method. The U-CIEDP methodology is centred on the end user and developed with the user’s interests at heart. Specifically, the U-CIEDP model consists of several small loops that allow for system refinement based on user feedback. Unlike the U-CIEDP approach, the READY method does not go through the entire process at once. Instead, it integrates each element after it meets the target features. This can be seen, for example, in Fig. 3, Step 5,7 & 8 where the simulator is depicted in a recursive loop with the objective of producing a synthetic dataset based on user feedback. In this way, the developed system is tested and validated by the user at each stage of development. Following the development of the READY method, five users were invited to test each component of the system at Middlesex University’s Smart Space Lab.

A major goal of this section is to evaluate the READY method by gathering feedback from end level users. To achieve this, professionals from different industries were invited to join the evaluation process. The system evaluation was conducted online due to COVID-19 travel restrictions. The evaluation approach was structured as follows.

Twelve external participants were involved in the evaluation process. Although the participants came from different industries, they were all involved, in some capacity, with smart homes. Five of the users were executives of smart home automation companies; five were care assistants; and two were researchers in the smart home domain. The system assessment process took place online. Prior to completing the assessment, the author created a consent form using Qualtrics^1^ and sent a link to each user so they could give their consent. After consent was provided, a Zoom^2^ link was distributed so each user could join an online and watched demonstration of the READY system.

Two sets of questionnaires were supplied to the participants during the assessment. The first section of the questionnaires was issued before the demonstration of the system, while the second section of the questionnaires was issued after the demonstration. The questionnaire can be found in [2]. The assessment session took 40–60 min in total.

#### Results

The first part of the questionnaires (Q1 & Q2) measured the participants’ knowledge of smart homes. Over 90% of participants demonstrated an exceptional understanding of smart home technology. Moreover, 80% of participants were aware of the advanced capabilities of smart homes, such as detecting health emergencies and diseases and providing advice on lifestyle changes. In spite of these high levels of smart home knowledge at baseline, all of the participants (i.e., 100%) believed that their knowledge of smart home services improved (Q5) after viewing the demonstration.

The next part of the questionnaires (Q3) measured the importance of user involvement in home automation. More than 80% of participants said it was extremely important to provide user guidance about how and when automation services should be performed.

Next, the questionnaires (Q4) measured the usefulness of the smart home system. All of the participants (i.e., 100%) believed it was vital to ensure that users had access to smart home services as soon as they moved into their home. This belief underscores both the need for and the value of the READY system, which was created with the intent of providing users with smart home services right from the outset.

Finally, the questionnaires touched on how effective the system was in carrying out the user’s desired activities. As mentioned, U-CIEDP is a system development method in which the user has an opportunity to provide input at every step of the process. This is done to decrease the likelihood of user complaints in the future. Indeed, survey responses showed that 95% of the participants in the evaluation felt confident that the system was able to effectively carry out the user’s desired activities (Q6). One participant, however, felt unsure of this without being able to personally use the system.

### Discussion of the evaluation

As with the evaluation of the U-CIEDP method, the evaluation of the READY method involved a number of key stakeholders. This evaluation involved having participants observe a demonstration of the system online and subsequently provide feedback. The evaluation was based on three core criteria needed for a successful system-immediacy, personalisation and effectiveness. Each of these is discussed in turn below.

#### Immediacy

Providing services right away was a key objective of the project. This need was confirmed in the evaluation, as all 12 participants strongly agreed that the system should start working as soon as a person moves into the home. The five home care assistants, in particular, stressed that placing an elderly person in a smart home that does not provide services instantly could put the occupant at risk. Relatedly, a few participants argued that that user acceptance would be limited if smart home services could not be initiated as soon as the user started living in the house. Although the READY system is capable of providing basic home automation as soon as a user moves in, future research should focus on adding advanced smart home services such as fault detection, anomaly detection and other related services. This would likely have a positive impact on user acceptance.

#### Personalisation

Personalisation was another key objective of the project. READY uses the U-CIEDP approach where the user is at the core of system development. The READY approach begins with an interview with the user for the purpose of programming initial user requirements. Future system refinements, however, come directly from user feedback. In this way, the system both comes and remains personalized to its specific user. In reviewing survey responses, 100% of the participants believed that a user would be able to assist the developer in personalising the system. However, 80% of the participants expressed uncertainty over users’ abilities to successfully control the automation process on their own. This is likely because a large majority (i.e., 80%) of those who participated in the evaluation were end-level users, meaning they may not have had the necessary depth of knowledge to make an informed judgement. However, the two participants who did have an intricate knowledge of smart home systems-the smart home researchers-agreed that the user would be able to independently control over the automation process.

#### Effectiveness

The third criterion of importance was creating a system that could actually provide the services needed by the user. The READY approach was designed to be flexible, consisting of several loops which allow the system to be refined based on user input. With each successive piece of feedback, the system adapts, enabling it to become more effective at meeting user requirements. Importantly, this process treats the user as an active participant in the system development process, offering the user a sense of agency in adding or modifying the smart home features they most need. As a case in point, after the user has been living in the smart home for a short period of time, the user is asked if the system is working for them as desired. If the user has any concerns or desired changes in mind, then the rules of the system can be modified. In this way, the READY method keeps the user in the loop until the system is operating at a maximum efficiency. Nearly all the participants in the evaluation validated this point, with 95% expressing confidence that the READY system could effectively provide home services.

## Conclusions

This paper comprehensively reports the requirements elicitation, design and validation of a tailored, smart home system capable of providing smart home services to a new user as soon as they start living in the house.

We designed the system to incorporate feedback and validation from frequent user interactions. This approach is consistent with the User-Centric Intelligent Environment Development Process (U-CIEDP) framework. The project integrated four approaches namely, survey, simulation, activity recognition and transfer learning, together with using the useR-guided nEw smart home Adaptation sYstem (READY) method.

We used a survey to identify user expectations and behaviour before their first contact with the system. The simulation processed user responses to the survey and converted them into data. Finally, we used this data to design a smart home services solution for the user. Thus, we validated both the developmental method and the designed solution in a real scenario. Further, we introduced the User-guided Transfer Learning (UTL) method, supplementary to the READY method, which involved users in the design of enhancements to the automation process.

Five users test the complete system at the Smart Spaces Lab in Middlesex University. Almost all of the users agreed that the smart home should provide automation services as soon as they start living in the home. Furthermore, over 80% of participants believed that our design system was able to provide the required services from day one.

Following this, twelve professionals attended an online event where the system is demonstrated. Feedback indicated that they found this sort of system beneficial to win the trust of new users. Additionally, they noted that traditional smart home companies offered the same product for everyone. Nevertheless, our approach provides a personalised system for the individual user, which interested them. So, they referred to design several smart home features, which could be part of future research.

In our project, UbikSim, LFPUBS, and MReasoner were considered because they were developed based on the ECA (Event-Condition-Action) paradigm. Hence, the output produced is human-understandable, which provide critical support to the system development in the initial stage. By using these tools, system development was possible with much less effort than had we developed our own tools. We noted, however, that more accurate results may have been possible to achieve had we chosen to develop these tools ourselves.

Although we focused on a same user system adaptation, in principle our approach can also be used with data and rules generated from a different user. However, the number of iterations required to converge into satisfaction will be proportional to how similar the habits and technology are in the source data and rules.

The next step of this project will be to design a more automated system that enables the tools to communicate more directly without less need for human intervention or further data processing. Increasing participant involvement in system validation will also open up new challenges.

## Footnotes

1 https://www.qualtrics.com.

2 www.zoom.com.
